# 3D vs. 2D simulated fetoscopy for spina bifida repair: a quantitative motion analysis

**DOI:** 10.1038/s41598-023-47531-9

**Published:** 2023-11-28

**Authors:** Mirza Awais Ahmad, Yolan Weiler, Luc Joyeux, Elisenda Eixarch, Tom Vercauteren, Sebastien Ourselin, Jan Deprest, Emmanuel Vander Poorten

**Affiliations:** 1 Department of Mechanical Engineering Sciences, Catholic University of Leuven, 3000 Leuven, Belgium; 2 Obstetrics and Gynaecology, University Hospital of Leuven, 3000 Leuven, Belgium; 3 BCNatal Fetal Medicine Research Center, Hospital Clinic, Hospital Sant Joan de Déu, Universitat de Barcelona, Barcelona, Spain; 4 Institut d’Investigacions Biomèdiques August Pi i Sunyer (IDIBAPS), Centre for Biomedical Research on Rare Diseases (CIBERER), Barcelona, Spain; 5 Department of Imaging and Biomedical Engineering, Kings College, London, WC2R 2LS UK

**Keywords:** Biomedical engineering, Endoscopy, Three-dimensional imaging, Statistics

## Abstract

3D imaging technology is becoming more prominent every day. However, more validation is needed to understand the actual benefit of 3D versus conventional 2D vision. This work quantitatively investigates whether experts benefit from 3D vision during minimally invasive fetoscopic spina bifida (fSB) repair. A superiority study was designed involving one expert team ($>18$ procedures prior) who performed six 2D and six 3D fSB repair simulations in a high-fidelity animal training model, using 3-port access. The 6D motion of the instruments was recorded. Among the motion metrics are total path length, smoothness, maximum speed, the modified Spectral Arc Length (SPARC), and Log Dimensionless Jerk (LDLJ). The primary clinical outcome is operation time (power 90%, 5% significance) using Sealed Envelope Ltd. 2012. Secondary clinical outcomes are water tightness of the repair, CO$_2$ insufflation volume, and OSATS score. Findings show that total path length and LDLJ are considerably different. Operation time during 3D vision was found to be significantly shorter compared to 2D vision ($113\pm 9$ vs. $149\pm 19$ min; p $=$ 0.026). These results suggest enhanced performance with 3D vision during interrupted suturing in fetoscopic SBA repair. To confirm these results, a larger-scale follow-up study involving multiple experts and novice surgeons is recommended.

## Introduction

Stereoscopy is an imaging technology enabling three-dimensional (3D) vision. It uses two cameras that capture the same scene from two different viewpoints, similar to humans, where images from both eyes, situated at an offset, perceive depth. If the left/right images from the stereo camera are “displayed” exclusively to the corresponding left/right eye, the human visual cortex can similarly interpret the different viewpoints and perceive depth in the stereo images.

This study investigates whether 3D imaging is useful for minimally invasive fetoscopic surgery (MIS). Spina bifida repair serves as a case study. Related literature investigating the benefits of 3D visualization reported that passive polarised glasses produce more conclusive results (in favor of 3D) in laparoscopic interventions^1^. In 3D, complex tasks can be executed more easily and with less stress^2–4^, and operation time and error reduced^5^. Better surgical performance and lower stress are reported^6^, whereas reduced tool path length and lower stress measured via NASA’s Task Load Index^7^. Many studies comparing 3D and 2D endoscopy showed significant differences by using these outcome metrics, however, *motion metrics* could provide an additional and possibly more objective assessment. This work analyses the surgeon’s motion in fetoscopic spina bifida repair allowing for a truly objective assessment of performance and skill.

Spina bifida (SB) aperta is a congenital neural malformation that represents about 10% of all spinal deformities causing physical and mental impairment which require intense lifelong medical follow-up^8^. It is caused by incomplete closure of the neural tube, leading to exposure of neural elements to the amniotic fluid. This causes trauma and damage to the spinal cord and nerves, resulting in motor function loss of the limbs^9^. Treatment options are either postnatal or prenatal repair. Prenatal repair halts and prevents ongoing neurodegenration and has been shown to improve the outcome, as demonstrated in the management of myelomeningocele study (MOMS) trial^10^. In that study, repair was done through laparotomy and hysterotomy and layered anatomical repair, referred to as open fetal surgery (OFS). To avoid complications from hysterotomy, like uterine rupture in subsequent pregnancies, significant efforts have been made to make this operation minimally invasive (MIS) using fetoscopic instrumentation^11^.

Prenatal MIS fetoscopic repair may reduce maternal and fetal risks, however, an MIS approach is more challenging than its open counterpart. The vulnerable surgical environment and difficult closure techniques are combined with common disadvantages of endoscopic approaches: the fulcrum effect that inverts motion, the reduction of motion to only four degrees of freedom, poor tactile feedback, loss of depth perception, and difficult hand-eye coordination together leading to increased operation times. 3D imaging techniques could help the surgeon regain the sense of depth and improve efficiency.

This work exploits quantitative motion metrics to objectively assess whether 3D fetoscopy actually benefits expert surgeons in performing prenatal MIS for fSB-repair. Our hypothesis is that the skills of expert surgeons improve when performing fetoscopic repair with 3D vision rather than 2D vision and that these improvements are measurable using quantitative motion metrics.

## Methods

### Study design

A superiority study was designed involving one expert laparoscopic fetal surgeon (JD) and assistant (LJ) performing a simulated fetoscopic SB repair in a high-fidelity animal training model using 3-port access^12^. The primary outcome of this experiment was total operation time (TOT) which includes the preparation of the animal model until the end of the last step of the surgery. Based on estimated TOT, six surgeries per group (2D vs. 3D) were required $(\beta =90\%, \alpha =5\%)$. Secondary clinical outcomes were water tightness at the end of the repair, total volume CO2 used, and OSATS score. To document the performance of the surgeon in terms of instrument use, 39 motion metrics were computed including path length, smoothness, maximum speed, the modified Spectral Arc Length (SPARC), Log Dimensionless Jerk (LDLJ), Number of Speed Peeks (NSP) and Number of Acceleration Peeks (NAP)^13–20^. These metrics are calculated for the surgeon’s left hand (LH), right hand (RH) and the assistant’s hand holding the fetoscope (FH).

Since clinically, today’s fetoscopic surgeons would typically transit from 2D to 3D vision^21,22^, the surgeon first operated in 2D after which all 3D experiments were performed. Prior to the first operation herein, the surgeon conducted 18 interventions on the model. An earlier study using competency cumulative sum (C-CUSUM) analysis^12^ demonstrated competency after six consecutive procedures.

### Training model

The experiments used a high-fidelity training model as earlier described by Joyeux et al.^12^. In an adult male New Zealand rabbit animal model all operative steps and surgical conditions present in current clinical multi-layered fetoscopic spina bifida repair, can be simulated. The rabbit’s abdominal cavity mimics the available intra-uterine workspace (15 $\times$ 10 $\times$ 5 cm) when insufflated with 3 l, of CO$_2$ at a pressure of 5 mmHg^23^. The back of the fetus (22–24 weeks of gestation) is represented by the rabbit stomach^24^. In this model, laparoscopic gastric Nissen fundoplication^25^ the ten surgical steps required for fetoscopic spina bifida repair, including gastric wall patch suturing, can be mirrored, and necessitate delicate tissue handling, thorough dissection, hemostasis, and suturing.

### Surgical procedure

We used adult New-Zealand rabbits and standard pediatric laparoscopic instruments and a 3D endoscope (Karl Storz™ 4 mm scope, TIPCAM1 S, 7240 BA3D). Four main motion sequences were identified containing similar movements to fetoscopic repair. These sequences are used for comparison of 3D vs. 2D motion-based skill:

- I. Dissection movements: the surgeon performs a series of explorative, dissection and grasping movements to free up the esophagogastric junction (EGJ) in preparation of the fundoplication.
- II. High-force movements: the surgeon creates an anti-reflux valve by pulling the upper part of the stomach (called the fundus) behind the EGJ.
- III. Interrupted suturing: the surgeon performs the fundoplication with a series of interrupted sutures.
- IV. Running sutures: the surgeon sutures a patch on the large anterior curvature of the rabbit stomach with two semi-circular running sutures.

The secondary outcomes included the individual operation time for motion sequences I, II, III, and IV, and their sum which equals the fetal operation time (FOT) and the time spent on operative steps, while the TOT includes the preparation and conclusion steps as well.

**Table 1:** Metrics used for quantitative motion analysis.

Metric	Equation
Total time	$T_i \triangleq \int _{t_{i,start}}^{t_{i,end}}dt = t_{i,end}-t_{i,start}$
Path length	$P_i \triangleq \int _{0}^{T_i}v_i(t)dt$
Volume	$$V_i = $$ convhull($$p_i(t)$$)
Maximum speed	$S_{i,max} \triangleq max(v_i(t))$
Mean speed	$\bar{S}_i \triangleq mean(v_i(t))$
Speed consistency	$\sigma _{S_i} \triangleq std(v_i(t))$
Number of speed peaks	$NSP_{i,\alpha }\!\!\triangleq \left| \{v_i(t)\,|\,\frac{dv_i(t)}{dt}\!\!=0,\!\frac{d^2v_i(t)}{dt^2}<0\!,\!v_i(t)\!>\!\alpha \bar{S}_i\!\}\!\right|$
Speed peak rate	$SPR_{\alpha ,i} = \frac{NSP_{i,\alpha }}{T_i}$
Movement arrest period ratio	$MAPR_{i,\alpha } \triangleq \frac{T_{i,v_i(t)>(\alpha /100)S_{i,max}}}{T_i}$
Maximum acceleration	$A_{i,max} \triangleq max(a_i(t))$
Mean acceleration	$\bar{A}_i \triangleq mean(a_i(t))$
Acceleration consistency	$\sigma _{A_i} \triangleq std(a_i(t))$
Number of acceleration Peaks	$NAP_{i,\alpha } \triangleq \left| \{a_i(t)\,|\,\frac{da_i(t)}{dt}=0,\, \frac{d^2a_i(t)}{dt^2}<0,\,a_i(t)>\alpha \bar{A}_i \} \right|$
Acceleration peak rate	$APR_{i,\alpha } = \frac{NAP_{i,\alpha }}{T_i}$
Integral of acceleration vector	$IAV_i \triangleq \int _{0}^{T_i}a_i(t)dt$
Smoothness	$Sm_i \triangleq \frac{1}{T_i}\sqrt{\frac{1}{2}\int _{0}^{T_i}\left[ \left( \frac{d^3x}{dt^3}\right) ^2 + \left( \frac{d^3y}{dt^3}\right) ^2 + \left( \frac{d^3z}{dt^3}\right) ^2\right] dt}$
Spectral arc length	${SAL_i \triangleq -\int _{0}^{\omega _c}\sqrt{\left( \frac{1}{\omega _c}\right) ^2 + \left( \frac{d\hat{V}_i(\omega )}{d\omega }\right) ^2}d\omega ,}$ ${\hat{V}_i(\omega )=\frac{\hat{V}_i(\omega )}{V_i(0)}}$
Modified spectral arc length	$SPARC_i\!\!\!\!\triangleq \!\!\!\! -\int _{0}^{\omega _c}\sqrt{\left( \frac{1}{\omega _c}\right) ^2 + \left( \frac{d\hat{V}_i(\omega )}{d\omega }\right) ^2}d\omega , \hat{V}_i(\omega )\!\!=\frac{\hat{V}_i(\omega )}{V_i(0)}\,,\, \omega _c \triangleq min \left\{ \omega _c^{max} \,|\, min\{\omega \,|\,\hat{V}_i(r)<\bar{V}_i, \forall \,r > \omega \} \right\}$
Dimensionless jerk	$DLJ_i \triangleq \frac{(T_i)^3}{v_{i,peak}^2} \int _{0}^{T_i}j_i(t)^2dt$
Log dimensionless jerk	$LDLJ_i \triangleq ln\left| DLJ_i \right|$
Visibility ratio	$v_i \triangleq \frac{T_{motion,i}}{T_{i}}$

The $\left| \,.\, \right|$ denotes the cardinal of a set and *i* denotes the metric corresponding to the *i*th segment. The function convhull() is a built-in MATLAB^®^ (Mathworks, Massachusetts, US) function.

### Experimental setup

**Figure 1:**
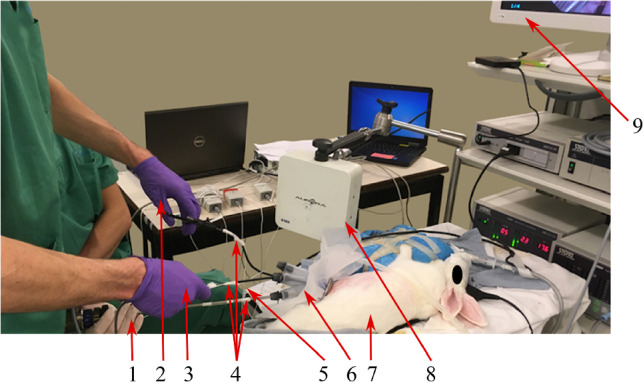
Set-up of experiment. (1) Assistant’s hand holding the fetoscope. (2) Left hand (LH) of expert surgeon holding a tool. (3) Right hand (RH) of expert surgeon holding a tool. (4) Aurora sensors taped to LH tool, RH tool, fetoscope. (5) Operating table corner used as a reference for Aurora tracker. (6) The fetoscope port with aurora sensor attached. (7) The male adult New Zealand rabbit. (8) Aurora tracker magnetic field generator (FG). (9) The display showing images from the fetoscope.

Figure 1 shows the experimental setup. The Aurora (NDI, Ontario, Canada) electromagnetic field generator (FG) henceforth referred to as the Aurora tracker, measures the 3D position and 3D orientation of the LH tool, the RH tool, the fetoscope, and the fetoscope port. All instruments had a dedicated electromagnetic tracking (EMT) sensor attached. Because the Aurora tracker has only four input ports, only four sensors can be tracked simultaneously. Therefore, during some instrument changes sensors needed to be dis- and reconnected. Upon instrument switch or when a surgical step or phase was completed, Aurora recordings were stopped. This results in each trial being segmented in multiple motion recordings. Notes were made during the surgery to keep track of the recorded segments or unexpected events. When non-connected instruments were used briefly, the connections were not changed. This results inevitably in motion recordings having partially invisible (sensor out of range) segments.

The endoscope used in this study was a Karl Storz™ 4 mm scope (TIPCAM1 S, 30° angle, 18 cm length, 7240 BA3D) which allows to easily switch from 2D to 3D vision^26^. To perform 3D surgery, we used the same HD monitor and operators were wearing the Karl Storz™ 3D glasses. Unlike autostereoscopy^27^, the HD monitor allows multiple users to have a stereo view as long as they wear the 3D glasses. During surgery, one surgical assistant manipulated the fetoscope while the expert surgeon performed the operation. Seven laparoscopic instruments were used. Video recordings of the full surgical scene and fetoscope were made.

### Motion metrics and clinical outcomes

Motion can be analyzed to assess the surgeon’s performance under different circumstances^14^. Table 1 lists the different motion metrics that were employed alongside the equations to calculate them. The metrics were computed for the LH, RH and fetoscope, and port motions. The SPARC, LDLJ, path length and maximum, mean, and consistency of the speed and acceleration are time-independent motion metrics while the others are time-dependent. Time independence is an important characteristic for a metric to truly represent skill.

The EM sensors may have been only visible for a duration of time that is relevant for a specific segment of the procedure. For this, the visibility ratios $v_{i}$ are calculated to provide a metric for the reliability of the motion data. Here, the motion data is considered “visible” even if one of the three (RH, LH, and fetoscope) instrument’s EM sensor measurements are available for a relevant surgical step.

### Motion data processing

**Figure 2:**
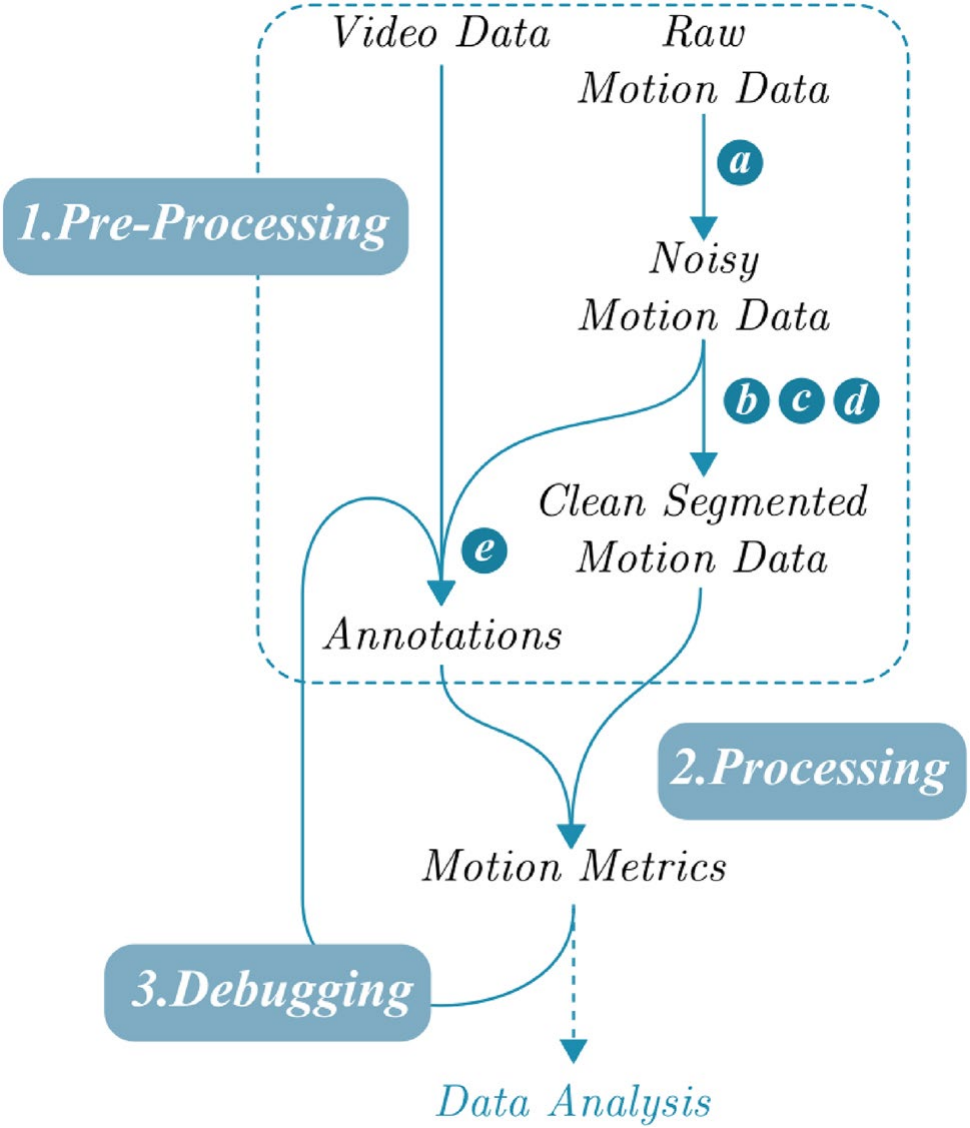
Data processing schematic. (**a**) Formatting, (**b**) transformations, (**c**) visibility, (**d**) smoothing and (**e**) annotations. The pre-processing, processing and debugging steps are performed using MATLAB^®^.

Both the motion and video data are used in the process to calculate the motion metrics. Figure 2 shows the process with three main steps: pre-processing, processing, and debugging. These steps are computed with MATLAB^®^. The goal of pre-processing is to transform the raw motion data captured from the experiment into motion data that is suited for calculating the motion metrics. Pre-processing can be further split into five separate steps:

- Formatting (a) data in the right structure. The stored data files (encrypted ROS bag files) from the Aurora tracker is transformed into a readable database.
- Transforming (b) the data to the correct reference frame. A corner of the operating table (number 5 in Fig. 1) is used as the base reference frame for all sensors.
- Pruning (c) invisible data. Motion data that was invisible for more than 1 second is removed from the data to not affect the metrics. Segments that were invisible for less than a second are filled using quadratic spline interpolation based on the visible data points before and after the invisible segment.
- Smoothing (d) the data. A butterworth filter ($f_c=10$ Hz, $n=5$) and an exponential filter ($\alpha =0.05$) are applied to the motion data.
- Annotating (e) the data. Starting and ending timestamps of relevant surgical step segments in motion data are noted manually based on the fetoscope’s video.

Processing consists of calculating the required motion metrics for each of the four global motion sequences mentioned above. In order to do this, it is necessary to aggregate the results of the smaller motion segments *i* generated during step c. Depending on the motion metric, aggregation is done differently. For example, the path length $P_i$ of each segment is summed together to represent the total path length of the sequence, whereas the mean speed $\bar{S}_i$ of each segment is averaged to find the overall mean speed. The maximum speed $S_{i,max}$ is aggregated by taking the maximum speed of all segments. The last step, named debugging ensures that the quadratic interpolation adopted to process the invisibility is performed accurately: If the interpolation generated a maximum acceleration above 6 ms$^{-2}$, which is outside the range of human-produced accelerations, this part of motion data is not included in the analysis .

### Statistical data analysis

The null hypothesis states that the means of the motion metrics obtained in the 3D group are equal to those in the 2D group. This requires an unpaired t-test or, in the case the metrics were not normally distributed or when the variances $\sigma ^2$ were not equal, a parametric Wilcoxon rank-sum test. To test for normality and equal variances we used the Saphiro-Wilk test and the F-test respectively. The software GraphPad Prism 8.4.2. (Dotmatics, Boston, MA, USA) was used for processing these tests.

### Ethical approval statement

This experiment was approved by the Animal Ethics Committee of the Group Biomedical Sciences of the KU Leuven (P093-2016). The experiments were done following the ARRIVE guidelines for animal research^28,29^, and the guidelines of the National Centre for the Replacement, Refinement and Reduction of Animals in Research (NC3Rs).

## Results

### Clinical outcomes

For each clinical outcome the mean for both the 2D ($\mu _{2D}$) and 3D ($\mu _{3D}$) groups are shown in Table 2, as well as their difference $\mu _{(2D-3D)}$. A 95% confidence interval (CI) is reported as well. All execution times are lower when using 3D and TOT, as well as FOT, are significantly reduced in the 3D group with an average reduction of respectively 35 and 24 minutes. Taking into account the different steps in the surgery, the operation time for the dissection movements (I) and interrupted suturing (III) show an even more obvious difference. For the dissections, a 40% reduction is appearing, and the time for interrupted suturing is reduced from an average time of 21.7 minutes in the 2D group to 13.2 minutes in the 3D group, which is more than a 30% reduction. No differences are found for the OSATS score nor for the insufflation volume.

**Table 2:** Unpaired t-test for clinical outcomes.

Outcome	$\mu _{2D}$	[95% CI]	$\mu _{3D}$	[95% CI]	$\mu _{2D-3D}$	[95% CI]	P value
OSATS (/25)	18.5	[15.8, 21.2]	19.3	[15.8504, 22.7]	−.833	[−5.83, 4.16]	0.718
$CO_2$ Vol. (l)	255	[165, 345]	184	[118, 250]	71.2	[−55.2, 198]	0.238
OT I: Dissection motions	30.2	[16.8, 43.6]	17.8	[14, 21.6]	12.3	[7.15, 40.8]	0.001**
OT II: High-force motions	10.5	[6.44, 14.6]	8.83	[6.05, 11.6]	1.67	[10.8, 59.9]	0.521
OT III: Interrupted suturing	21.7	[12.88, 3.52]	13.2	[1.6912, 15.7]	8.5	[5.04, 12]	<0.001***
OT IV: Running suturing	56.2	[52.2, 6.24]	54.7	[45, 59.4]	1.5	[−9.89, 12.9]	0.775
OT I–IV: Foetal	119	[117, 12.7]	94.5	[88.2, 10.8]	24	[−3.92, 7.25]	0.011*
Total OT incl. model preparation	149	[130, 168.5]	113	[104, 122]	35.3	[6.04, 18.6]	0.026*

$\alpha =0.05$, *$p\le 0.05$, **$p\le 0.01$, ***$p\le 0.001$, bold: Wilcoxon rank sum test instead of unpaired t-test.

*OT* operation time.

### Quantitative motion outcomes

#### Visibility

The visibility ratios of the motion recordings are shown in Table 3. Sequences III and IV are well recorded with an average visibility of respectively 91% and 94%. Sequences I and II are less well recorded due to missing recordings and lower visibility. The bold values show visibility below 80%, suggesting the motion data is less reliable.

**Figure 3:**
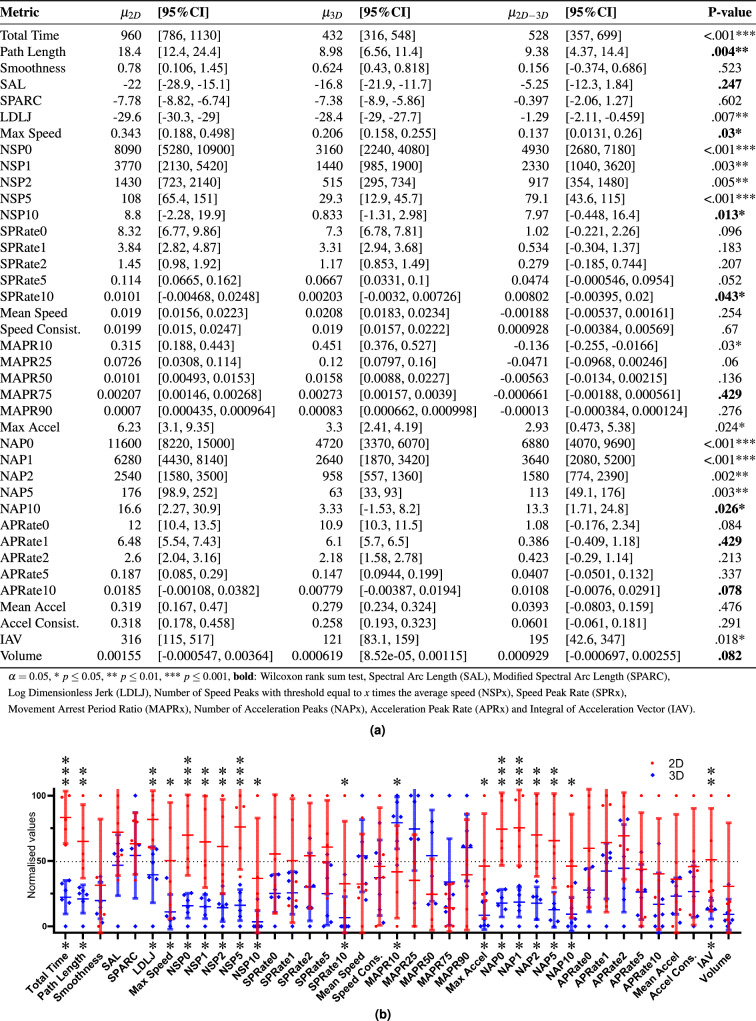
Right hand motions during interrupted suturing comparing 3D (blue) and 2D (red) vision. $n_{2D} = 5, n_{3D} = 6$.

**Figure 4:**
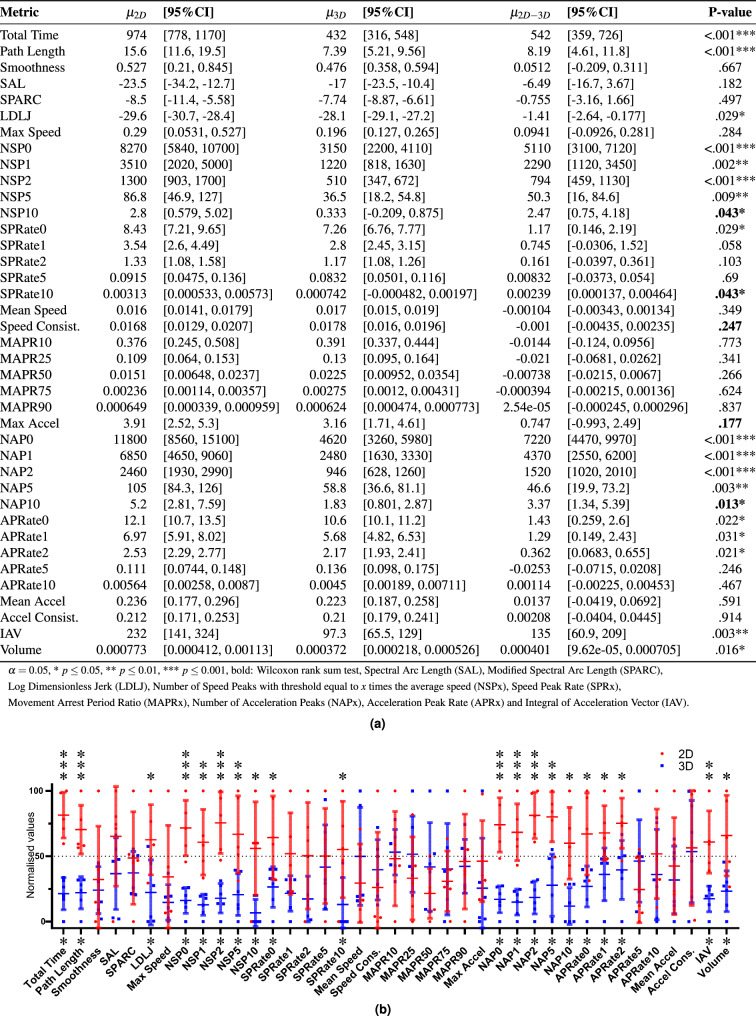
Left hand motions during interrupted suturing comparing 3D (blue) and 2D (red) vision. $n_{2D} = 5, n_{3D} = 6$.

Recordings for trial 3 for the 2D group as well as a segment in trial 5 for both 2D and 3D groups are missing due to technical issues. The asterisk (*) marks a potential mismatch between motion signals and surgical movements due to the difficulties with the annotations. In these cases the motion segment was chosen to be slightly longer than the actual movement on the video, to make sure that the motions typical for the surgical procedure are also included in the final sequence. The downside of this approach is that motions not representative of relevant surgical step might get introduced as well, reducing the data quality. Instead of a visibility ratio greater than 1, the visibility is set to be equal to the average visibility of that sequence.

**Table 3:** Visibility (%) for all trials.

Trial	1		2		3		4		5		6	
Motion Sequence	2D	3D	2D	3D	2D	3D	2D	3D	2D	3D	2D	3D
I: Dissections	82.1	**69.5**	81.5	98.6	0	99.6	**74.1**	88.1	86.6	0	**68.2**	90.2
II: High force motions	87.1	98.4	86.5	88.5*	0	88.5*	91.4	**79.6**	0	**76.9**	81.3	99.1
III: Interrupted suturing	91.3	90.8	89.9	91.4*	0	86.7	96.1	99.1	81.4	98.9	94.4	81.5
IV: Running suturing	97.5	99.6	94.0	94.7	0	90.2	91.1	91.9	85.9	99.9	95.6	91.5

*Visibility calculated differently than explained in chapter 3. Bold: visibility below 80%.

Another way to check the reliability of the motion data is to compare the total motion time from Figs. 3 and 4 with the clinical fetal operation time from Table 2. Indeed, the previously reported clinical operation time is showing a considerable difference for the dissection motions (I) and interrupted suturing motions (III). For the latter, the motion-based total time is also demonstrating a considerable difference, confirming the clinically measured operation time. In the case of the dissection motions, the motion-based total time is showing a considerable difference for the right hand but not for the left hand, even though the left and right hand are equally used. This contrasts with the clinically measured operation time and therefore suggests that the motion data of this sequence is not totally reliable. The visibility analysis confirms these findings. Therefore only detailed results are shown in the following for the interrupted suturing motions.

#### Motion metrics

The results of the statistical analysis comparing the motion metrics from the 3D and 2D groups are reported in Table 4. More detailed data for the right hand and left hand for the interrupted suturing motions are reported in Figs. 3 and 4. The surgeon’s left hand has the Acceleration Peak Rate (APR) as an additional significantly different metric. Note that the total time shown in these tables is calculated using the motion data of the corresponding instruments rather than the clinically measured operation time.

There are no significant differences in the running suturing motions (IV) while multiple considerable differences are found when comparing interrupted suturing motions (III). The significant metrics for both surgeon’s hands and the assistant’s hand include path length, Log Dimensionless Jerk (LDLJ), Number of Speed Peaks (NSP), and Number of Acceleration Peaks (NAP). For the NSP and NAP metrics, the P-value is increasing with an increasing threshold $\alpha$. At a higher threshold, the motion metrics are often not deemed normally distributed. Therefore a non-parametrical Wilcoxon-ranksum test instead of a student t-test is used. These metrics are marked in bold in the tables. The SPARC, Smoothness, MAPR, SPR and APR are not showing significant differences.

## Discussion

### Principal findings

Results indicate significant differences in total operation time, fundoplication time, and dissection time between 3D and 2D fetoscopy, in line with available literature. This suggests that 3D vision makes fundoplication and dissections easier. A significant difference was found for several quantitative motion metrics such as path length, Log dimensionless jerk, number of speed peeks, and number of acceleration peaks when comparing interrupted suturing. No significant differences were detected when running suturing motions were compared. Highly standardized tasks like running sutures may over time become automatic for experienced surgeons, negating immediate benefits of 3D fetoscopy^30^.

### Results in existing knowledge context

Several studies have looked at the differences in skill under 3D and 2D vision between novices or experts. In these, differences in operation time were found for running suturing tasks^14–17^. In our case, running suturing motions (IV) *does not* show quantitative differences. Motion analysis has been subject to a high degree of variability from study to study. Patel et al.^31^ compared 2D and 3D fetoscopy’s impact on spina bifida repair using a low fidelity simulator. Surgical performance was gauged subjectively through skin cutting, dural patch placement, and suturing, using the NASA-TLX questionnaire^32^. In 16 participants there were no significant differences except for 3D’s shorter total operation time. In a study by Wilhelm et al.^33^, experienced laparoscopists among 48 study participants, excelled with a 3D endoscope. Operation time and instrument path length improved with 3D, but there was no difference in NASA-TLX scores. Nomura et al.^34^ compared imaging modes for endoscopic submucosal dissection in animals, finding comparable operation times. 3D reduced time and negatively impacted eyestrain symptoms (excluding blurred vision), as measured on a 100 mm visual analog scale^35^.

The SPARC, as proposed in^18^, is showing a non-significant improvement towards the 3D group. Although the metric is known to be time-independent and consistent such as in the detection of Parkinson’s disease^19^, it is sensitive to noise and therefore requires the right tuning in order not to be affected too much. We assume that the interrupted suturing sequence that we have been using for the comparison includes too many different motions for this metric to appear decisive in our comparison. We expect that it would be necessary to further segment the four motion sequences into smaller more distinguishable movements to leverage this metric to allow for stronger conclusions. The LDLJ, another advanced and recurring motion metric in literature, does show a noticeable difference, although small. This suggests a detectable motion difference between 3D and 2D vision during interrupted suturing. As for the SPARC, our whole analysis could possibly be improved as well by further splitting the motion data.

The threshold $\alpha$ is slightly affecting the motion metrics NSP, NAP, and their corresponding SPRate and APRate. It appears that the metrics with a higher threshold are not normally distributed and become less suitable for comparison. Although this group of metrics is showing very significant differences for both the right and left hand ($P_{NSP0,NAP0}=<0.001$), due to their nature they are highly dependent on the total motion time. Therefore they do not provide much additional information in addition to the motion time.

### Clinical and research implications

Given the progressive nature of spina bifida, ensuring a watertight closure of the dura and fascia layers over the neural placode is critical to prevent amniotic fluid-related damage^36^. Various closure methods, like suturing, can significantly influence repair quality. Through subtask-level motion analysis, this study directly gauged the impact of stereo fetoscopy on these tasks. This approach could potentially unveil alternative closure techniques for better outcomes or optimize existing methods by leveraging specific imaging technology.

During interrupted suturing motions (III), many significant differences are found. During this surgical step, four interrupted sutures are required in order to perform the fundoplication. In contrary to the running suture technique, a knot is required for every suture. The surgeon has to perform more manipulations with the suture. Moreover, the dynamic range of movements also increases; instead of a continuous motion making the semi-circular suture, the surgeon has to move the fundus to perform the suture at a totally different location. Because the manipulations take place in the full range of the surgical field, it appears that improved depth perception is needed for successful completion. A similar interpretation could be made for the dissection motions (I), where the surgeon has to manipulate a series of soft tissues laying caudal in the rabbit. The surgeon has to extend the tools far in the rabbit to reach the tissues, also requiring more depth perception. Such surgical tasks provide an interesting opportunity to be used as a training or evaluation exercise to study the effects of 3D vision vs. 2D vision on the skill level of a surgeon.

**Table 4:** P values for unpaired t-tests comparing the motion metrics.

Metric	I: Dissection movements			II: High-force movements			III: Interrupted Suturing			IV: Running suturing		
	RH	LH	Scope	RH	LH	Scope	RH	LH	Scope	RH	LH	Scope
Total time	0.004**	0.523	0.094	0.345	0.114	0.345	<0.001***	<0.001***	<0.001***	**0.792**	0.849	0.858
Path length	0.019*	0.29	0.077	0.29	0.065	0.245	0.004**	<0.001***	0.016*	0.641	0.613	0.422
Smoothness	0.87	0.786	0.97	**0.257**	**0.999**	0.773	0.523	0.667	0.18	0.063	0.086	0.073
SAL	0.234	0.968	**0.151**	0.983	**0.999**	0.708	**0.247**	0.182	0.222	0.222	0.077	0.173
SPARC	0.876	0.346	0.246	0.882	0.545	0.322	0.602	0.497	0.996	0.764	0.054	0.935
LDLJ	0.187	0.238	0.395	0.812	0.506	0.766	0.007**	0.029*	0.011*	0.081	0.076	0.228
Max speed	0.145	0.045*	0.557	0.718	**0.25**	**0.352**	0.03*	0.284	**0.429**	**0.247**	0.21	0.199
NSP0	0.016*	0.674	0.074	0.125	0.066	0.17	<0.001***	<0.001***	<0.001***	0.46	**0.999**	0.261
NSP1	0.056	0.86	0.135	0.113	0.046*	0.224	0.003**	0.002**	0.002**	0.976	0.682	0.255
NSP2	0.011*	0.524	0.095	0.127	0.071	**0.352**	0.005**	<0.001***	0.001**	0.616	**0.537**	0.515
NSP5	0.023*	0.229	0.063	0.111	0.484	0.24	<0.001***	0.009**	0.004**	0.129	0.555	0.785
NSP10	0.016*	0.754	0.5	**0.81**	**0.429**	0.038*	0.013*	0.043*	0.035*	0.061	**0.515**	0.932
SPRate0	**0.095**	0.477	0.387	0.005**	0.22	0.119	0.096	0.029*	0.061	0.239	0.529	0.06
SPRate1	**0.421**	0.396	0.73	0.033*	0.233	**0.914**	0.183	0.058	0.077	0.972	0.59	0.084
SPRate2	**0.151**	0.941	0.652	0.048*	0.651	0.968	0.207	0.103	0.502	0.543	**0.662**	0.409
SPRate5	0.114	0.123	0.159	0.644	0.083	0.778	0.052	0.69	0.328	0.118	0.526	0.792
SPRate10	0.032*	0.743	0.34	**0.81**	**0.786**	**0.105**	0.043*	0.043*	**0.238**	0.069	**0.537**	0.896
Mean speed	0.054	0.606	0.759	0.565	0.637	0.826	0.254	0.349	0.447	0.368	0.383	**0.662**
Speed consist.	0.448	0.058	**0.999**	**0.61**	**0.571**	**0.476**	0.67	**0.247**	**0.429**	0.033*	0.971	0.418
MAPR10	0.159	0.853	0.492	0.725	0.78	0.551	0.03*	0.773	0.617	0.224	0.481	0.201
MAPR25	0.31	**0.905**	0.345	0.672	0.676	0.441	0.06	0.341	0.76	0.796	0.39	**0.126**
MAPR50	0.31	0.483	0.239	0.462	0.373	0.995	0.136	0.266	0.926	0.431	0.284	**0.126**
MAPR75	0.192	0.465	**0.421**	0.447	0.463	0.803	**0.429**	0.624	0.11	0.108	0.11	**0.247**
MAPR90	0.236	0.309	0.565	**0.476**	0.852	0.707	0.276	0.837	**0.931**	0.046*	0.093	**0.329**
Max accel	0.01*	0.583	0.481	0.422	0.088	0.886	0.024*	**0.177**	**0.537**	0.128	**0.931**	**0.792**
NAP0	0.01*	0.641	0.06	0.17	0.081	0.199	<0.001***	<0.001***	<0.001***	0.546	0.699	0.348
NAP1	0.029*	0.736	0.072	0.174	0.076	0.224	<0.001***	<0.001***	0.002**	0.441	0.561	0.573
NAP2	0.015*	0.429	0.087	0.17	0.102	0.61	0.002**	<0.001***	0.004**	0.921	**0.429**	**0.999**
NAP5	0.019*	0.269	0.539	0.417	0.668	0.701	0.003**	0.003**	0.054	0.319	0.36	0.975
NAP10	0.024*	0.177	0.493	0.895	0.21	**0.905**	0.026*	0.013*	0.17	0.2	0.634	**0.429**
APRate0	**0.095**	0.372	0.285	0.005**	0.323	0.081	0.084	0.022*	0.059	0.261	0.479	0.072
APRate1	0.55	0.439	0.28	0.024*	0.036*	0.362	**0.429**	0.031*	0.107	0.426	0.408	0.471
APRate2	0.461	0.595	0.876	0.082	0.969	0.466	0.213	0.021*	0.926	0.909	**0.429**	0.883
APRate5	0.171	0.332	0.468	**0.999**	0.09	0.969	0.337	0.246	0.406	0.357	0.411	0.979
APRate10	0.208	0.209	0.289	0.784	**0.786**	**0.714**	**0.078**	0.467	**0.623**	**0.429**	0.656	**0.429**
Mean accel	0.548	**0.905**	0.628	0.354	0.163	0.374	0.476	0.591	0.141	0.295	**0.329**	0.177
Accel consist.	0.378	0.504	0.844	**0.171**	0.586	0.712	0.291	0.914	0.203	0.208	0.891	0.199
IAV	0.035*	0.419	**0.151**	0.074	0.024*	0.138	0.018*	0.003**	0.009**	0.388	**0.662**	0.25
Volume	0.051	0.511	0.117	**0.61**	0.044*	**0.352**	**0.082**	0.016*	**0.177**	0.104	**0.537**	0.926
P count	14	1	0	5	4	1	18	20	12	2	0	0

$\alpha =0.05$, *$p\le 0.05$, **$p\le 0.01$, ***$p\le 0.001$, bold: Wilcoxon rank sum test.

*SAL* spectral arc length, *SPARC* modified spectral arc length, *LDLJ* log dimensionless jerk, *NSPx* number of speed peaks with threshold equal to *x* times the average speed, *SPRx* speed peak rate, *MAPRx* movement arrest period ratio, *NAPx* number of acceleration peaks, *APRx* acceleration peak rate, *IAV* integral of acceleration vector.

Robot-assisted surgery’s growing popularity prompts exploration of its application in spina bifida repair. Many available surgical robots feature console stereo displays. Falk et al.^3^ evaluated 3D visualization’s impact using the Da Vinci telerobotics system. Metrics included time, task errors, and kinematic analysis. Results demonstrated the superiority of 3D over 2D at the same/higher resolution, enabling faster, more precise performance. Given the delicate nature of fetal spina bifida repair, robotic solutions would offer stable instruments and high resolution 3D vision. Supported by this study, this makes them an encouraging consideration for surgical teams worldwide.

### Strengths and limitations

In this study, we have underscored the significance of pre-processing raw data and introduced methods for that. Also, this study has undertaken a more detailed analysis of surgical steps by breaking them down into specific actions and maneuvers at sub-task level, subsequently annotating the data accordingly. Additionally, we have conducted a comprehensive exploration of 39 motion metrics extracted from existing literature to determine which metrics are most conducive to providing meaningful comparisons in future investigations.

An important limitation of this research is the low number of simulations performed (sample size $n_{2D} = n_{3D} = 6$) while comparing the impact on just one expert endoscopic-fetal surgeon. This study however was powered to detect differences in operation time (primary outcome) and not in motion metrics. Furthermore, technical challenges, such as sensor breakage, workspace being moved out of range during procedure, recording system crash resulted in a further reduction of the motion tracking data, making the data for dissection (sequence I) and high-force movements (sequence II) unreliable. In case of technical errors in the motion tracking system and loss of signals, the trial was not interrupted in order not to increase operation time and animal suffering.

Interested surgeons may face some challenges in replicating this study due to ethical, logistical and financial hurdles linked to animal trials. To address this concern and further improve the external validity by increasing trial numbers and surgeons, one could adopt high or low fidelity surgical simulators, including for spina bifida repair^31,37,38^.

## Conclusion

In the high-fidelity rabbit model for fetoscopic SBA repair, 3D vision via 3-port access substantially shortens operation time for an expert fetal surgeon during interrupted suturing. A substantial difference was found for several quantitative motion metrics such as path length, Log dimensionless jerk, number of speed peeks, and number of acceleration peaks. No substantial differences were detected when running suturing motions were compared.

Three takeaways with respect to motion analysis for future research are summarised here. Firstly, this research shows that one can draw interesting conclusions from comparing interrupted and running suturing motions. Secondly, the importance of time-independent motion metrics, like SPARC, has been pointed out. It is indeed important to have different motion metrics based on multiple aspects. The SPARC and LDLJ are two metrics that have been consistently performing in other literature under the right conditions which is why the recommendation is made to define the sample size based on this metric. The challenge will be to segment the motion data up to a sufficiently small resolution. Finally, it is extremely important to record the full surgical scene and to provide cues in the video when the motion recording is started or stopped. This provides major help in annotating the motion recordings such that accurate and precise motion segments can be used for comparison.

This research opens the door for follow-up studies in the field of 3D vs 2D motion analyses. The results should be interpreted from a guidance perspective. It is a pilot study that demonstrates the urge and potential for research on this topic and has proposed key learnings to help future research.

## Data Availability

The datasets generated during and/or analyzed during the current study are available from the corresponding author upon reasonable request.
